# Detecting intermittent switching leadership in coupled dynamical systems

**DOI:** 10.1038/s41598-018-28285-1

**Published:** 2018-07-09

**Authors:** Violet Mwaffo, Jishnu Keshavan, Tyson L. Hedrick, Sean Humbert

**Affiliations:** 1 Department of Mechanical Engineering, University of Colorado, Boulder, CO USA; 2 Department of Biology, University of North Carolina at Chapel Hill, Chapel Hill, NC USA

## Abstract

Leader-follower relationships are commonly hypothesized as a fundamental mechanism underlying collective behaviour in many biological and physical systems. Understanding the emergence of such behaviour is relevant in science and engineering to control the dynamics of complex systems toward a desired state. In prior works, due in part to the limitations of existing methods for dissecting intermittent causal relationships, leadership is assumed to be consistent in time and space. This assumption has been contradicted by recent progress in the study of animal behaviour. In this work, we leverage information theory and time series analysis to propose a novel and simple method for dissecting changes in causal influence. Our approach computes the cumulative influence function of a given individual on the rest of the group in consecutive time intervals and identify change in the monotonicity of the function as a change in its leadership status. We demonstrate the effectiveness of our approach to dissect potential changes in leadership on self-propelled particles where the emergence of leader-follower relationship can be controlled and on tandem flights of birds recorded in their natural environment. Our method is expected to provide a novel methodological tool to further our understanding of collective behaviour.

## Introduction

The self-organizing structure observed in biological and physical systems such as bacterial colonies^1^, fish schools^2^, bird flocks^3^, smart mob^4^, and mechanical oscillators^5^ are attractive for their potential application in science and engineering. These structures can be extended to social and engineered systems^6^ and consist of interacting units that can be represented as a network where the nodes are individuals and the edges represent the links between them. The network graph representation of such complex structure is essential to understand and anticipate the mechanisms underlying the emergence of collective behaviour^7,8^ as well as their resilience to noise and external perturbations^9^.

The study of the interaction between individuals using a networked approach is pervasive across science as illustrated by the large attention of researchers in the field of biology^10^, neuroscience^11^, epidemiology^12^, social sciences^13^, and technology^4^. Particular attention has been devoted to the notion of leadership which is defined^14^ as the initiation of new heading directions by some individuals which are then followed by other group members. In several complex systems, such notion is thought to be fundamental in explaining the emergence of collective behaviour and thus central to the understanding of the interaction between group members. In biological groups for example, it has been reported that leadership might benefit the group in foraging^15^ or avoiding predatory attacks^16,17^.

Several approaches have been proposed to quantify causal relationships in coupled dynamical systems using time series based observational datasets. These methods may rely on an underlying model or be model-free. Examples of model based methods include cross-correlation^18^ and Granger causality^19^ which have been successfully utilized to dissect causal directional relationships in biological groups^14,20^, physical systems^21,22^, and in social science^23^. In such methods, it is hypothesized that the parameters of a system can be well apprehended through linear approximation of system dynamics^8^.

In general, the dynamics of real world systems might be subject to complexities including consistent noise perturbations, varying time delays, and nonlinearities^24,25^. In such cases, model-free methods such as event synchronization^26^, and those based on information-theoretic measures^27–29^ ranging from transfer entropy^30^, conditional transfer entropy^31^, maximum entropy^32^, causation entropy^33,34^, and union transfer entropy^35^ offer the possibility of dissecting causal relationships without the assumption of an underlying model nor linearity. Event synchronization has been successfully applied to dissect causal relationships in complex atmospheric phenomena^36^ and policy diffusion^37^. Transfer entropy has been applied to predict cause effect relationships in animal robot interactions^38,39^, predator-prey interactions^40,41^, and neuronal and physical networks^29,42^.

To address the limitations inherent to individual methods in dissecting between leader and follower, we have demonstrated in^43^, that a combination of individual methods yields more robust and consistent results. Yet, identifying leadership based on current approaches poses several challenges. Specifically, in prior works, the fundamental hypothesis assumes that group leaders are consistent in time and in space which may not hold^44,45^ since in a large group undergoing complex manoeuvres, for example, the group might experience an instantaneous change of its heading direction resulting in a change of group leadership. In addition, it is commonly assumed in existing approaches that the dynamics of group leaders are completely independent from the rest of the group. This might not be realistic since in the absence of consensus^46^, leadership might also be influenced by a subset of followers. Also, the study of groups with multiple leaders has received less attention for the reason that they might possibly induce large correlation lengths and cluster formation^47^.

To the best of our knowledge, few works have addressed the question of intermittent switching leadership. Recently, two methods based on Granger causality and implemented on data segments were utilized to reveal intermittent causal relationship in between the observation length^48,49^. Another method build on time series analysis has been proposed in^50^ to dissect spatio-temporal propagation patterns in leader-follower relationships. In this work, we develop a novel method to detect switching and intermittent leadership in coupled networked dynamical systems. Our approach builds on non-linear and model-free measures of causality: transfer entropy or directed event synchronization. Our algorithm initially determines appropriate data partitioning by systematically increasing the length of the detection window starting from a small interval allowing the computation of the causality measures to an optimal value. Then from the network divergence which quantifies the level of influence of each individual on other group members assessed using either of these measures, it computes the cumulative influence of individuals on other group members over successive time windows. A change in the cumulative influence of an individual apprehended through these indicators is interpreted as a change in its causal relationship. Such changes occur when the cumulative influence over consecutive data segments reaches either a maximum or exhibits a significant decrease in slope, when a leader switches roles to become a follower, or passes through a minimum or displays a significant increase in slope, when a follower takes leadership.

We test our approach initially on synthetic datasets which allow us to control the emergence of leadership^43^. Self-propelled particle models such as the Vicsek model^51^ offer an excellent mathematical framework for generating such datasets and allow testing of the resilience of the method to variation of noise level, strength of the interaction between particles, and density of particles which might considerably affect the level of coordination of the group^3^. Next, we apply our approach to a data-driven model of fish shoaling^52,53^ which has recently emerged as an excellent testbed for replicating real world fish datasets departing from experimental data. Data-driven models offer the possibility to recreate realistic locomotory pattern of animal as observed in their natural environment and incorporating detailed individual dynamics along with specific interactions rules^52–60^. Finally, we demonstrate our method on real world datasets of bird flocks observed in the nature^61^. These data consist of trajectories of tandem flight of cliff swallows (*Petrochelidon pyrrhonota*) engaged in a chase pursuit. Using our method, we seek to unravel any change in the causal relationship over the period of observation as opposed to traditional methods which consist of assessing leadership over the entire length of the dataset.

## Materials and Methods

### Detecting causal relationships in networks of dynamical systems

We consider the case of a complex system whose states can be apprehended through *N* time series ${\{{x}_{t}^{(i)}\}}_{t=1}^{T}$, where *t* is the time step, *T* is the total length of the time series, and *i* = 1, …, *N* represent individuals or components of the system. We seek to uncover leader-follower relationships within the group by studying the influence of each individual on other group members. Similar to^43^, we assimilate the system to a directed network where individuals are the nodes and the links are measured by the strength of the interaction between them determined by weighted directed edges.

A network structure can be depicted by a graph of *i* = 1, …, *N* finite nodes denoted $G=({\mathscr{V}}, {\mathcal E} ,{A}_{(\cdot )})$ with set of nodes ${\mathscr{V}}$ of cardinality *N*, set of edges ${\mathcal E} ={\mathscr{V}}\times {\mathscr{V}}$, and adjacency matrix *A*^(⋅)^ with elements ${A}_{(\cdot )}^{(ij)}\ge 0$, where _(⋅)_ indicates the method to identify interaction. For out-diagonal elements, ${A}_{(\cdot )}^{(ij)}=0$ if no interaction exist between nodes *i* and *j*, otherwise ${A}_{(\cdot )}^{(ij)} > 0$. For convenience, diagonal elements are set to ${A}_{(\cdot )}^{(ij)}=0$. We distinguish undirected networks where the adjacency matrix *A*^(⋅)^ is symmetric, that is ${A}_{(\cdot )}^{(ij)}={A}_{(\cdot )}^{(ji)}$ for *i* ≠ *j*, and directed network when there exist *i* and *j* such that ${A}_{(\cdot )}^{(ij)}\ne {A}_{(\cdot )}^{(ji)}$, meaning that node exerts a strong influence on another node. Note also that for fixed or static networks, the interaction between nodes is constant over time, that is ${A}_{(\cdot )}^{(ij)}={A}_{(\cdot )}^{(ji)}$, whereas in networks with switching on-off topology, ${A}_{(\cdot )}^{(ij)}=0$ when no interaction exists between *i* and *j*, otherwise ${A}_{(\cdot )}^{(ij)}={A}_{(\cdot )}^{(ij)}(t) > 0$. We comment that, in the above formulation, only pairwise interaction is considered whereas in practice, one might observe triplets where two nodes interact indirectly through a third node^33^. To investigate leader-follower relationships, we consider a threshold value $\bar{A} > 0$ such that for two nodes *i* ≠ *j*, *i* influences *j* if and only if ${A}_{(\cdot )}^{(ij)} > \bar{A}$, otherwise ${A}_{(\cdot )}^{(ij)}=0$. In practice, such a threshold value can be determined by a significance test such as a t-test implemented on a sampled or shuffled surrogate datasets where no interaction exists between time series depicting node dynamics^62^. In particular, the t-test returns the t-confidence interval for the sample average value $\bar{X}$ with confidence level 1 − *α* determined as $\bar{X}\pm t\frac{\varsigma }{\sqrt{n}}$ where *ς* is the standard deviation of the sample, *n* is the sample size, *t* is the t-distribution critical value with *n* − 1 degree of freedom and significance level *α*. Note that most software returns the values of the confidence interval whose upper bound can be selected as the threshold value.

We construct the directed interaction between individuals using the network divergence which for convenience in our analysis of leader-follower is defined as the difference between nodes out-degree and in-degree, that is:

1 $${\rm{\Delta }}{S}_{(\cdot )}^{(i)}={S}_{{\rm{out}}}^{(i)}-{S}_{{\rm{in}}}^{(i)}=\sum _{j=1}^{N}{A}_{(\cdot )}^{(ji)}-\sum _{j=1}^{N}{A}_{(\cdot )}^{(ij)},$$

where ${S}_{{\rm{out}}}^{(i)}$ is known as the weighted out-degree and indicates the intensity of the influence of nodes *i* on others nodes; and ${S}_{{\rm{in}}}^{(i)}$ is known as the weighted in-degree and indicates the intensity of the influence of other nodes on nodes *i*. Therefore, $\Delta {S}^{(i)} > 0$ can be interpreted as individual *i* exerting more influence on the rest of the network nodes, while Δ*S*^(*i*)^ < 0 indicates that *i* is influenced by other nodes. Hence, we consider group leaders as individuals that exert a stronger influence on other group members quantified by ${\rm{\Delta }}{S}^{(i)} > 0$ and the problem of detecting leadership reduces to identify individuals *i* maximizing Δ*S*^(*i*)^. To assess ${\rm{\Delta }}{S}_{(\cdot )}^{(i)}$, we compute pairwise interaction between group members. Note that the approach can be easily extended to dissect the network graph in the case of multivariate time series^63^ and to account for multiple interactions^33,64^. Pairwise interactions ${A}_{(\cdot )}^{(ij)}$ are quantified between nodes through two different measures namely transfer entropy (TE)^27–30^ and directed event synchronization (ES)^26,36^ which have been successfully utilized in the literature to diagnose non-linear causal relationships between time series. Although the method is illustrated using transfer entropy and event synchronization, other relevant measures of causality capturing complex form of interactions such as triplets can also be utilized. This include causation entropy^33,34^ and spike synchronization^37^.

### Transfer entropy

Transfer entropy (TE)^30^ from individual *j* to *i* measures the reduction in the uncertainty in predicting ${\{{X}_{t}^{(i)}\}}_{t}$ given values of ${\{{X}_{t}^{(j)}\}}_{t}$ and is quantified through the expression:

2 $${{\rm{TE}}}^{(ji)}=\sum _{{{\chi }}^{3}}\,p({X}_{t+1}^{(i)},{X}_{t}^{(i)},{X}_{t}^{(j)})\mathrm{log}\,\frac{p({X}_{t+1}^{(i)}|{X}_{t}^{(i)},{X}_{t}^{(j)})}{p({X}_{t+1}^{(i)}|{X}_{t}^{(i)})},$$

where ${\{{X}_{t}^{(i)}\}}_{t}$ and ${\{{X}_{t}^{(j)}\}}_{t}$ are time series, considered as stationary process, depicting the dynamics of nodes *i* and *j* respectively, and taking values in a finite set ${\chi }$ allowing to estimate the probability and joint probability distributions *p*(⋅), *p*(⋅, ⋅), and *p*(⋅, ⋅, ⋅) either through histograms^65^ or kernel density estimators^30^; $p({X}_{t+1}^{(i)},{X}_{t}^{(i)},{X}_{t}^{(j)})$ is the joint probability of future and current states of individual *i* and *j* respectively; $p({X}_{t+1}^{(i)}|{X}_{t}^{(i)},{X}_{t}^{(j)})$ is the conditional probability of future states of *i* given current states of both *i* and *j*; and $p({X}_{t+1}^{(i)}|{X}_{t}^{(i)})$ is the probability of future states of *i* given its current state.

Note that TE^(*ij*)^ = TE^(*ji*)^ = 0 if and only if the processes ${X}_{t}^{(i)}$ and ${X}_{t}^{(j)}$ are independents implying that the probabilities $p({X}_{t+1}^{(i)}|{X}_{t}^{(i)},{X}_{t}^{(j)})$ and $p({X}_{t+1}^{(i)}|{X}_{t}^{(i)})$ are equals. Otherwise, ${{\rm{TE}}}_{ji} > 0$ indicates that information flows from individual *j* to individual *i*. Finally, the weighted directed influence of node *i* on node *j* measured by transfer entropy can be assessed against spurious causal relationships by comparing the values of net transfer entropy against a threshold ${\bar{A}}_{({\rm{TE}})}\ge 0$, that is

3 $${A}_{{\rm{TE}}}^{(ij)}\,:\,=\{\begin{array}{ll}{{\rm{TE}}}^{(ij)}-{{\rm{TE}}}^{(ji)} & {\rm{if}}\,{{\rm{TE}}}^{(ij)}-{{\rm{TE}}}^{(ji)} > {\bar{A}}_{{\rm{TE}}},\\ 0 & {\rm{otherwise}}\mathrm{.}\end{array}$$

### Directed event synchronization

Event synchronization (ES)^26^ measures the synchronicity between pairs of time series through the occurrence of events within a given time interval $\tau  > 0$. An event can be identified for example by a rapid change in an individual heading direction or a spike in the acceleration time series. To implement the method, the time series of events ${e}_{k}^{(i)}$ occurring at time ${t}_{k}^{(i)}$, *k* = 1, …, *m*^(*i*)^, where *m*^(*i*)^ is the number of events identified, is extracted for each time series ${\{{X}_{t}^{(i)}\}}_{t}$, *i* = 1, …, *N*. To evaluate synchronicity between events^66^, from the waiting time between events ${d}_{{k}_{i}{k}_{j}}={t}_{{k}_{i}}^{(i)}-{t}_{{k}_{j}}^{(j)}$, the dynamical delay is defined as $\tau =\frac{1}{2}\,\min ({d}_{{k}_{i}{k}_{i-1}},{d}_{{k}_{i}{k}_{i+1}},{d}_{{k}_{j}{k}_{j-1}},{d}_{{k}_{j}{k}_{j+1}})$ and is utilized to compute the directed influence between ${e}_{k}^{(i)}$ and ${e}_{k}^{(j)}$ as:

4 $${s}_{{k}_{i}{k}_{j}}=\{\begin{array}{ll}\min ({w}^{{k}_{i}},{w}^{{k}_{j}}) & {\rm{if}}\,0 < {d}_{{k}_{i}{k}_{j}}\le \tau ,\\ 0 & \,{\rm{otherwise}},\end{array}$$

where ${w}^{{k}_{i}}$ is a weight associated to the number of events identified at the same location *k*_*i*_ in ${\{{X}_{t}^{(i)}\}}_{t}$. Finally, defining *τ*_*max*_ as the maximum delay for two events to be considered synchronized, directed event synchronization between *i* and *j* is computed as:

5 $${{\rm{ES}}}^{(ij)}=\sum _{{k}_{i}=1}^{{m}^{(i)}}\,\sum _{{k}_{j}=1}^{{m}^{(j)}}{S}_{{k}_{i}{k}_{j}}\mathrm{.}$$

The above quantity is normalized between 0 to 1 to facilitate comparisons of the strength of the interaction in different trials.

Using a threshold value ${\bar{A}}_{({\rm{ES}})}\ge 0$ computed using a t-test on sampled surrogate datasets to discard spurious links, the directed event synchronization between *i* and *j*, can then be utilized to construct the weighted adjacency matrix measuring the interaction between individuals *i* = 1, …, *N* as

6 $${A}_{{\rm{ES}}}^{(ij)}\,:\,=\{\begin{array}{ll}{{\rm{ES}}}^{(ij)}-{{\rm{ES}}}^{(ji)} & {\rm{if}}\,{{\rm{ES}}}^{(ij)}-{{\rm{ES}}}^{(ji)} > {\bar{A}}_{({\rm{ES}})},\\ 0 & {\rm{otherwise}}\mathrm{.}\end{array}$$

### Detecting intermittent switching leadership

For each time series ${\{{X}_{t}^{(i)}\}}_{t\mathrm{=1}}^{T}$, *i* = 1, …, *N*, we decompose the entire length of the observation *T* into *t*_*win*_ = 1, …, *T*_*seg*_ segments of length *win* enabling the computation of transfer entropy and event synchronization. For each time window *t*_*win*_, we evaluate the directed influence of individual *i* on other group member through the network divergence ${\rm{\Delta }}{S}_{(\cdot )}^{(i)}({t}_{win})$ defined in Eq. (1) for *t*_*win*_ = 1, …, *t*_*seg*_, with *t*_*seg*_ ≤ *T*_*seg*_. These values are utilized to build a measure of the directed influence as time evolves by integrating over the time segments as:

7 $${S}_{(\cdot )}^{(i)}({t}_{seg})=\sum _{{t}_{{win}}=1}^{{t}_{seg}}{\rm{\Delta }}{S}_{(\cdot )}^{(i)}({t}_{win}\mathrm{).}$$

**Figure 1:**
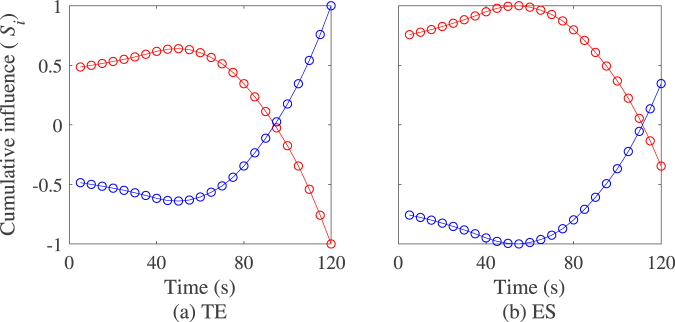
Illustration of the cumulative influence function estimated with transfer entropy (TE) and directed event synchronization (ES) on segmented raw datasets (circle) and interpolated dataset (solid line) overs the entire length of the observation. The plot in red indicates the influence of individual 1 on individual 2 and the plot in blue is the influence of 2 on 1.

The above quantity is a step function indicating the evolution of the directed influence for individual nodes measured at interval of length *win* after a number of time segments *t*_*seg*_ from the beginning of the observation period. Under the hypothesis that any change in the causal influence can be fully observed in between two time windows of length *win*, the best continuous and differentiable function approximating ${S}_{(\cdot )}^{(i)}$, that is ${\hat{S}}_{(\cdot )}^{(i)}(t)$, can be estimated using polynomial or spline interpolation. Prior to estimating ${\hat{S}}_{(\cdot )}^{(i)}(t)$, a filter is applied to ${S}_{(\cdot )}^{(i)}$ to smooth the function. Figure 1 illustrates the method with the values of ${S}_{(\cdot )}^{(i)}$ computed in subsequent data segments along with a spline interpolation ${\hat{S}}_{(\cdot )}^{(i)}(t)$ on the entire length of the dataset. We comment that values of ${\hat{S}}_{(\cdot )}^{(i)}(t)$ might not match exactly those of ${S}_{(\cdot )}^{(i)}$. If such difference is not critical to assess the influence of a given individual on another one, it might result in small difference in estimating the extrema of ${S}_{(\cdot )}^{(i)}$. Hence, such difference should be considered in analysing the numerical values of the switching time.

The monotonicity of ${\hat{S}}_{(\cdot )}^{(i)}(t)$ can be studied and its extrema computed using numerical differentiation to identify changes into the causal relationship from any individual *i* and the rest of the group members. Specifically, initially if an individual *i* is a leader, it switches from leader to follower at a given time instant ${t}_{{\rm{\max }}}^{(i)} < T$ if either the function ${\hat{S}}_{(\cdot )}^{(i)}(t)$ reaches a maximum at ${t}_{max}^{(i)}$ before decreasing, that is ${t}_{{\rm{m}}{\rm{a}}{\rm{x}}}^{(i)}={\rm{a}}{\rm{r}}{\rm{g}}{\rm{m}}{\rm{a}}{\rm{x}}\{{\hat{S}}_{(\cdot )}^{(i)}(t)\}$, or its slope significantly decreases. In the case where *i* transitions from follower to leader, the function ${\hat{S}}_{(\cdot )}^{(i)}(t)$ reaches either a minimum at ${t}_{{\rm{\min }}}^{(i)} < T$ before increasing, that is ${t}_{{\rm{m}}{\rm{i}}{\rm{n}}}^{(i)}={\rm{a}}{\rm{r}}{\rm{g}}{\rm{m}}{\rm{i}}{\rm{n}}\{{\hat{S}}_{(\cdot )}^{(i)}(t)\}$, or its slope significantly increases. If leadership is constant over the observation period, the function ${\hat{S}}_{(\cdot )}^{(i)}(t)$ is monotonic increasing while for followers *j* ≠ *i*, the function is monotonic decreasing.

In case of multiple individuals, the computational time required to analyse the monotonicity of the cumulative influence index ${\hat{S}}_{(\cdot )}^{(i)}(t)$ for $i=\mathrm{1,}\,N$, can be reduced by setting a threshold value corresponding to the *p* = 1 − 2/*N* quantile of the final value of ${\hat{S}}_{(\cdot )}^{(i)}(t=T)$ for $i=\mathrm{1,}\,N$ to differentiate potential leaders from followers. This procedure allows to study the monotonicity only for the most influential individuals such as multiple leaders which share common state. In addition, to establish the order of influence in case more than a single leader/follower is detected such as in triplets, the method can be successively reapplied to a subset of leaders/followers discarding the most/less influential individuals till the chain of influence is clearly established. Further, if group leaders have different states, one might observed the formation of clusters^47^ which can be detected by computing the clustering coefficient defined by:

8 $${C}^{(i)}=\frac{2{\xi }^{(i)}}{{k}^{(i)}({k}^{(i)}-\mathrm{1)}},$$

where *ξ*^(*i*)^ is the total number of links existing in node *i* and its *k*^(*i*)^ linked nodes. If an individual *i* is at the center of cluster, *C*^(*i*)^ is expected to be higher. Other elements of the cluster can be identified by implementing a simple machine learning algorithm such as the *k*-means clustering which minimizes the total intra-cluster variance defined by

9 $$\sum _{i=1}^{k}\,\sum _{{X}^{(j)}\in {{\mathscr{C}}}^{(i)}}{\rm{dist}}({X}^{(j)},{\mu }^{(i)}),$$

where dist is a distance measure; ${{\mathscr{C}}}^{(i)}$ is the i^th^ cluster, with *i* = 1, …, *k*; *μ*^(*i*)^ is the centroid of ${{\mathscr{C}}}^{(i)}$; and *X*^(*j*)^ is the state of individual *j*. To implement the *k*-means algorithm, *k* is set to the number of individuals identified having the largest value of ${{\mathscr{C}}}^{(i)}$.

**Figure 2:**
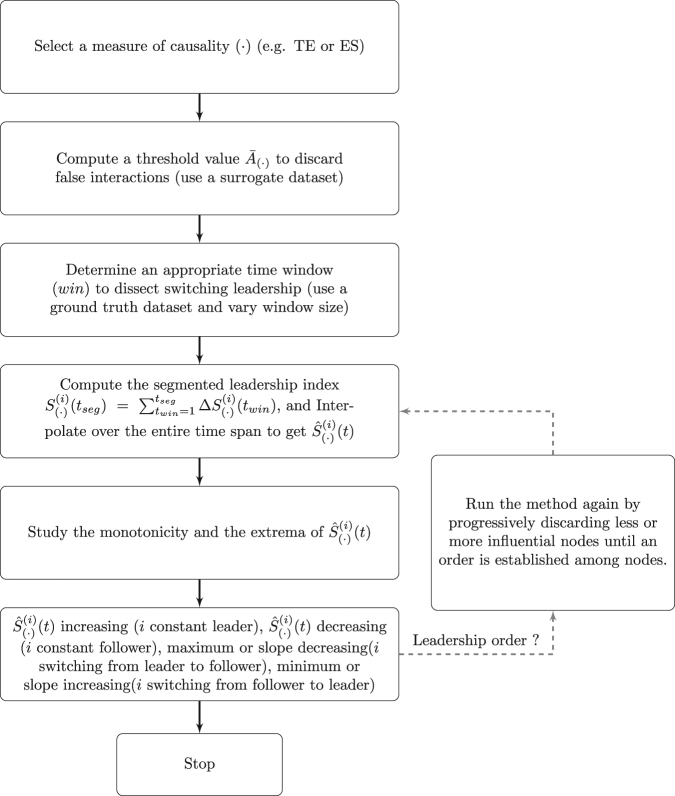
Steps to implement the method. The loop indicates the procedure to establish a leadership order in case of need.

To implement our method (see the flowchart in Fig. 2 and Supplementary Table S1 for further details), we hypothesize that data are sampled at a rate sufficiently large to allow good estimates of both event synchronization and transfer entropy in the small time interval *win*. In practice, the window length *win* is determined on a training dataset where switching leadership is well known. The training dataset can be generated using, for example, a theoretical model reproducing similar dynamics such as data-driven model of fish shoaling^43^ or by generating an artificial dataset from the segments of experimental data time series. An appropriate value of *win* can then be determined by initially setting a fixed value, and then increasing the window size until additional data points do not provide further information. In case of multiple switching events, the interpolation procedure might undermine the true value of the switching time. Hence, when a switching time is returned by the interpolated function, a correction can be introduced by taking instead the time corresponding to the peak value in the raw data closer to the estimated value of the extrema predicted by the interpolated function.

### Case study: triplets

**Figure 3:**
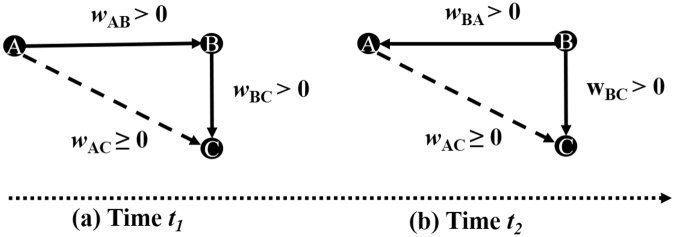
Illustration of the method on a triplet with directed interaction from A to B and B to C at a given instant *t*_1_, with leadership switching from A to B at another instant *t*_2_. Note that dashed line indicates whether the measures of causality are capable to detect or not the presence of direct/indirect interaction between A and C with either ${w}_{{\rm{AC}}} > 0$ or *w*_AC_ = 0.

To further illustrate the method, we consider at a time instant *t*_1_ the network configuration in Fig. 3(a) consisting of a triplet of the form A influences B and B influences C leading to an adjacency matrix with 0 everywhere except for the directed links from A to B, B to C, and eventually A to C with interaction weights ${w}_{{\rm{AB}}} > \mathrm{0,}\,{w}_{{\rm{BC}}} > 0$, and *w*_AC_ ≥ 0 respectively. Note that we do not consider self-loop. Also, in case of indirect interaction between A and C, the measures of causality might return either *w*_AC_ = 0 or ${w}_{{\rm{AC}}} > 0$. The network divergence in Equation 1 evaluated in the time interval *win* for node A, B, and C is Δ*S*_A_(*win*) = *w*_AB_ + *w*_AC_, Δ*S*_B_(*win*) = *w*_BC_ − *w*_AB_, and Δ*S*_C_(*win*) = −*w*_BC_ − *w*_AC_ respectively. Assuming the above values are sustained in all time windows, the cumulative influence index over successive time interval of length *win* will be ${S}_{i}={\rm{\Delta }}{S}_{i}(wi{n}_{1})+\ldots +{\rm{\Delta }}{S}_{i}(wi{n}_{{T}_{win}})$, for *i* = A, B, C, where *T*_*win*_ is the number of interval windows. Thus, *S*_A_ will be monotonic increasing and *S*_C_ will be monotonic decreasing. *S*_B_ will be monotonic increasing only if ${w}_{{\rm{BC}}} > {w}_{{\rm{AB}}}$, otherwise it will decrease or stay constant. An individual is identified as leader only if it has an increasing cumulative index otherwise it is a follower. From this consideration, A is a leader and C is a follower. Depending on the inequality ${w}_{{\rm{BC}}} > {w}_{{\rm{AB}}}$ or *w*_BC_ ≤ *w*_AB_, B will be identified either as a leader or as a follower. If both A and B are identified as leaders, then the order of influence can be determined by excluding C and repeating the analysis or simply by looking at the direction of the information flow between A and B. In both case, this will result in A identified as leading B. Similarly, if B is identified as follower, to determine the leadership order between B and C, A can be excluded from the analysis resulting in B leading C.

If at a different time instant *t*_2_, we consider that leadership switches from A to B, the rest remaining unchanged, then the network divergence in the time interval win for node A, B, and C will be Δ*S*_A_(*win*) = *w*_AC_ − *w*_BA_, Δ*S*_B_(*win*) = *w*_BA_ + *w*_BC_, and Δ*S*_C_(*win*) = −*w*_BC_ − *w*_AC_ respectively. The cumulative influence index for A, B, and C over successive time window can be computed as above. Since ${\rm{\Delta }}{S}_{{\rm{B}}}(win) > 0$ starting after time *t*_2_, *S*_B_ will either reach a minimum and start to increase, or its slope will significantly increase, indicating that B is taking leadership. *S*_C_ will keep decreasing since Δ*S*_C_(*win*) < 0. For A, *S*_A_ will either reach a maximum, then decreases or stays constant if *w*_AC_ ≤ *w*_BA_, or its slope will significantly decrease, indicating that A is switching to be a follower, otherwise it will increase indicating that A is also a leader. As above, the leadership order can be determined by discarding C and applying the method to A and B or simply by evaluating the direction of the information flow between A and B. Similar procedure can be applied to determine the leadership order between A and C.

### Modeling switching leadership in Vicsek model

The Vicsek self-propelled particle model (VM)^3,51^ proposes a discrete time framework to model collective behaviour in a group of *N* particles moving in a periodic bounded domain with constant speed *v*. This model can be augmented to include group leaders moving with constant heading angle ${\theta }_{0}^{(i)}$ while the rest of the group identified as followers update their heading angle ${\theta }_{t}^{(i)}$ by averaging closer neighbors orientation angle within a radius *R*^43^. For a particle *i* with position vector ${{\bf{r}}}_{t}^{(i)}={[{x}_{t}^{(i)}{y}_{t}^{(i)}]}^{{\rm{T}}}$, this updating rule is defined as:

10 $${U}_{t}^{(i)}=\{\begin{array}{ll}\frac{1}{|{{\mathscr{N}}}_{t}^{(i)}|}\sum _{j\in {{\mathscr{N}}}_{t}^{(i)}}{{\bf{v}}}_{t}^{(j)}, & {\rm{if}}\,{i}\,{\rm{is}}\,{\rm{a}}\,{\rm{follower}},\\ {{\bf{v}}}_{0}, & {\rm{if}}\,{i}\,{\rm{is}}\,{\rm{a}}\,{\rm{leader}},\end{array}$$

where ${{\bf{v}}}_{t}^{(j)}={v}{[\cos ({\theta }_{t}^{(j)})\sin ({\theta }_{t}^{(j)})]}^{{\rm{T}}}$ is the velocity vector of particle *j*; ${{\bf{v}}}_{0}={v}{[\cos ({\theta }_{0}^{(i)})\sin ({\theta }_{0}^{(i)})]}^{{\rm{T}}}$ is the leader’s constant velocity vector; ${{\mathscr{N}}}_{t}^{(i)}=\{j=\mathrm{1,}\ldots ,N:|{{\bf{r}}}_{t}^{(i)}-{{\bf{r}}}_{t}^{(j)}|\le R\}$ are the $|{{\mathscr{N}}}_{t}^{(i)}|$ particles found in a domain of radius $R > 0$ around particle *i*. Assuming particles are randomly distributed in a square bounded domain of length *L*, a number $K=1+\pi \frac{{r}^{2}}{{L}^{2}}(N-\mathrm{1)}$ of particles is likely to be within a circle of radius *R* of particle *i*^67,68^. Uncertainty in both leaders and followers heading angle is modeled by adding uniform noise to each individual heading direction.

The position ***r***^(*i*)^ and orientation *θ*^(*i*)^ of the *i*-th particle is determined in the Cartesian plane ${\mathbb{R}}$ as

11a $${x}_{t+1}^{(i)}={x}_{t}^{(i)}+v\,\cos ({\theta }_{t+1}^{(i)}),$$

11b $${y}_{t+1}^{(i)}={y}_{t}^{(i)}+v\,\sin ({\theta }_{t+1}^{(i)}),$$

11c $${\theta }_{t+1}^{(i)}={\rm{Angle}}[{U}_{t+1}^{(i)}]+\eta \zeta ,$$

where Angle[⋅] is a function returning the angle between the argument vector and the *x*-axis direction in the 2D plane; *η* ≥ 0 is the noise intensity; and *ζ* is uniform random noise with support in $[\,-\,\pi ,\pi )$.

We introduce switching leadership to the above model by intermittently assigning the leader or follower role to particles. Such assignment can be modeled through a deterministic process with scheduled single or repeated switches at identical or different frequencies. The network underlying the interaction in the Vicsek model given above is by definition time varying. Thus detecting switching leadership becomes more challenging since it requires differentiating between on-off links between particles and the larger causal influence induced by group leaders.

Using the Vicsek model, we generate synthetic datasets on group of *N* = 2 self-propelled particles. Particle positions and orientations are initially uniformly randomized within the square domain of length *L* = 1 and within the interval $[\,-\,\pi ,\pi )$ respectively. For each simulation, we perform 20,000 × ns + Ttr time steps where ns is the number of switching events and Ttr = 10,000 is a transient time for which simulations are discarded before the analysis. For a model with constant speed, a natural parameter to assess leadership in the Vicsek model is particle change of heading angle rate measured here by the turn rate and computed for the *i*-th particle using a simple first differentiation of the heading angle, that is ${\theta }_{t+1}^{(i)}-{\theta }_{t}^{(i)}$. Note that, in the analysis, transfer entropy is parametrized with a total number of 18 bins similar to^43^.

### Modeling intermittent switching leadership in fish shoaling

We test our method on a data-driven model designed to replicate fish shoaling behaviour as observed in experiments. The framework was first developed to capture the individual dynamics of barred flag-tails fish^54^ and later adapted to account for the specific locomotion of small fish species such as zebrafish^57^. In this model, the individual dynamic of *i* = 1, …, *N* fish at time *t* is captured by a mean reverting stochastic process^52,69^:

12 $$d{\omega }_{t}^{(i)}=v[-{\alpha }^{(i)}({\omega }_{t}^{(i)}-{}^{\ast }\omega _{t}^{(i)})dt+{\sigma }^{(i)}d{W}_{t}^{(i)}],$$

where ms^−1^ is the fish speed; ${\omega }_{t}^{(i)}\,({\rm{rad}}\,{{\rm{s}}}^{-1})$ is the turn rate; ${\alpha }^{(i)} > 0({{\rm{m}}}^{-1})$ measures the rate at which a fish engaged in a turning maneuver cease to turn; ${W}_{t}^{(i)}$ is a Wiener process with increments $d{W}_{t}^{(i)}$ defined as a Gaussian process with standard deviation $\sqrt{{\rm{d}}t}$; *v* is the constant speed; *σ*^(*i*)^(m^−1^ rad s^−1/2^) scales the level of uncertainty into fish heading direction; and ${}^{\ast }\omega _{t}^{(i)}({\rm{rad}}\,\,{{\rm{s}}}^{-1})$ is a response function.

Unlike the simple Vicsek model, the response function captures fish interaction with other fish by simulating alignment and attraction to closer neighbors and also interaction with the tank walls^52^. This function is modeled as:

13 $$\begin{array}{l}{}^{\ast }\omega _{t}^{(i)}={k}_{W}^{(i)}\frac{\mathrm{sign}({{\varphi }}_{W}^{(i)})}{{\tau }_{W}^{(i)}}+\frac{1}{N}\sum _{j=1}^{N}[{k}_{v}^{(i)}{v}^{(i)}\,\sin ({{\varphi }}_{t}^{(ij)})+{k}_{p}^{(i)}{d}_{t}^{(ij)}\,\sin ({\theta }_{t}^{(ij)})],\end{array}$$

where the first summand is the wall avoidance function with parameters the control gain ${k}_{W}^{(i)}$, the anticipated time to collision ${\tau }_{W}^{(i)}$, and the incidence angle with the wall ${\varphi }_{W}^{(i)}$; the second summand measures fish alignment with the rest of the group with parameters the alignment gain ${k}_{v}^{(i)}$ and the relative angle ${{\varphi }}_{t}^{ij}={{\varphi }}_{t}^{i}-{{\varphi }}_{t}^{j}$ of fish *i* with respect to fish *j*; the third summand controls fish grouping behaviour with parameters the attraction gain ${k}_{p}^{(i)}$, the difference in heading angle ${\theta }_{t}^{ij}$, and the distance ${d}_{t}^{ij}$ of a fish *i* with respect to another group member *j* ≠ *i*. Note that with the exception of parameters defining control gain, the rest is updated at each time step based on current and anticipated position and orientation of the fish with respect to the tank inertial frame.

Group leaders parameters are set to ${k}_{v}^{(i)}={k}_{p}^{(i)}=0$ while for followers, ${k}_{v}^{(j)} > 0$ and ${k}_{p}^{(j)} > 0$ *j* ≠ *i*. Switching leadership can be introduced in the model through a deterministic process following similar procedure as in the Vicsek model. Switching events can be assimilated to instances where the group drastically changes its swimming direction to avoid, for example, an obstacle or a predator attack.

The above model can be discretized under a standard Euler-Maruyama scheme to numerically compute the turn rate which can then be used to determine fish heading angle. The coordinates of fish position can also be determined using a simple Euler forward integration similar to the Vicsek model in Eq. (11). We comment that the above model can be viewed as a 2D representation of fish swimming in 3D such that there is no need for the model to incorporate collision avoidance between individuals in 2D. In fact, one can always suppose that trajectory crossing or overlapping represents a fish swimming above or below another fish. For 3D applications, the wall avoidance function can always be adapted to simulate collision avoidance between individual fish.

In the experiments, we generate 30 samples of the fish shoal model for groups of *N* = 2 fish including a leader using an Euler-Maruyama discretization scheme with time step 0.01 s. The model is simulated in a circular arena of diameter 4 m. Fish orientation is randomly initialized in the interval [−*π*, *π*) and positions are also uniformly randomized in the circular domain. Model parameters are summarized in Supplementary Information Table S2. In the fish shoal model, leader-follower relationship is obtained by setting ${k}_{p}^{(i)}={k}_{v}^{(i)}=0$ for group leaders and ${k}_{p}^{(j)} > 0$, ${k}_{v}^{(j)} > 0$ for the followers. Transfer entropy is evaluated on a total number of 18 bins similar to^43^.

### Experiments

#### Experiments on Vicsek self-propelled particles

The threshold value to differentiate true from false interactions is determined by generating a total of 30 sample datasets consisting of two non-interacting Vicsek particles for a total duration of 30,000 time steps. The first 10,000 time steps are discarded and we utilize transfer entropy and directed event synchronization to compute elements of the adjacency matrix ${A}_{(\cdot )}^{(ij)},i,j=\mathrm{1,}\,2$. The confidence interval of the mean value of ${A}_{(\cdot )}^{(ij)},i\ne j=\mathrm{1,}\,2$ is determined using a one-sided t-test at 5% confidence level. The upper bound is retained as the threshold value.

To identify an appropriate time window for switching leadership detection, a total of 30 sample Vicsek trajectories consisting of *N* = 2 particles and a single switching leadership event is generated. We set the initial switching detection time window to 100 time steps and run the algorithm to detect the predefined switching event. Then the time window is increased successively by adding an additional 100 time steps and repeating the same procedure to detect the switching event up to a time window of 2,000 time steps.

Using the selected threshold value and the switching detection time window, we test the method on Vicsek data in a series of four experiments. For each experiment, we generate a total of 30 samples of 2 Vicsek particles including a single leader and a single follower at a time for a total duration of 30,000 time steps and discarding the first 10,000 time steps. Initially, leadership is assigned to individual 1 and then switches to individual 2 after 10,000 time steps. Note that in case more than a single switching event is simulated, the number of time steps is increased by a factor of 10,000 for each event. In the case no switching is simulated, the simulations stop after 20,000 time steps.

In the first experiment, we simulate 30 samples of a group of two Vicsek particles with a single and constant leader over the entire length of the experiments. In the second set of experiments, we generate a total of 30 samples of a group of two Vicsek particles with leadership switching from individual 1 to 2 at a predefined time step. In the third experiment, we generate 30 samples of Vicsek particles with leadership switching twice, the first time from individual 1 to 2 at a predefined time steps 10,000, and the second time from individual 2 to 1 at time step 20,000 of the experiments.

In the fourth experiment, we test the resilience of our algorithm to varying levels of noise. We recall that in the Vicsek model, the level of coordination between agents is measured by the polarization defined by ${{\rm{P}}}_{t}=|{\sum }_{i\mathrm{=1}}^{N}\frac{{v}_{t}^{(i)}}{|{v}_{t}^{(i)}|}|$. When all agents move in the same direction, this order parameter takes value 1, and as noise in increased in the system, a phase transition from order to complete disorder is observed where the polarization tends to 0^3,51^. As such, the Vicsek model offers an excellent framework to test the resilience of our method to noise.

In the fifth experiment, we test the sensitivity of the method to multiple switching events at different frequencies by generating 30 sample datasets of *N* = 2 Vicsek particles including 2 switching leadership occurring after 10,000 time-steps each, one after 15,000 time-steps, and 2 occurring after 5,000 time-steps each. Finally, we test the method to group of more than two individuals in a series of three experiments. In the sixth experiment, we test the sensitivity of the method to triplets by simulating 30 sample of Vicsek particles where in average, each particle interacts with a single neighbour. In the seventh experiment, we generate 30 samples of *N* = 10 Vicsek particles including two leaders that share the same goal and in the eight experiment we simulate 30 datasets consisting of *N* = 10 Vicsek particles with a single switching event between two particles.

In all experiments, the accuracy of the method is measured by the ratios of true positive (TPR) and false positive (FPR). To illustrate the performance of the method for increasing noise level, we plot a receiver operating characteristic curve (ROC) parametrized by the noise intensity level varying from 0 to 1 in 0.1 increments. We recall that in a standard ROC curve, the points in the plot are parametrized by different cut-off values. In the ROC plot, when a method provides good classification of a binary test, the points in the ROC curve fall on the top left panel of the plot indicating high TPR and low FPR. However, when they fall within the diagonal plotted with dashed lines, the classifier performs poorly and the predictions are completely random.

#### Experiments on fish shoaling

Similar to the Vicsek model above, prior to implement the method, a threshold value to differentiate between true and spurious interactions is determined by generating 30 pairs of non-interacting fish datasets for 60 seconds. We compute pairwise interaction ${A}_{(\cdot )}^{ij},i,j=\mathrm{1,}\,2$ between fish using transfer entropy end directed event synchronization.

An appropriate time window segment to identify switching leadership in fish shoaling dataset is determined by generating 30 datasets of a couple of fish shoaling for a duration of 120 seconds including a single leadership switching event after 60 seconds. We then vary successively the detection time window from 1 to 10 seconds in increment of 1 second.

We consider for the fish shoaling three experiments where 30 sample datasets of pair of fish are generated including a single experiment with a constant leader, and two experiments with respectively single and multiple switching leadership. The datasets are generated for a duration of 70 seconds in case of no switching event and the duration is eventually increased by 60 seconds proportionally to the number of switching events predefined. This result in a duration of 130 seconds for the datasets with a single switching event, and 190 seconds for the datasets with two switching events. Prior to performing the analysis, we discard the first 10 seconds.

#### Tandem flights of cliff swallows

We implement our method to investigate potential change in leadership in tandem flights of birds drawn from a previously published paper^61^. Note that no animal use protocol was required since the videos were recorded in nature with no direct interaction with or handling of the animals. These data comprise 39 tandem flights recorded in the field using three high-speed video cameras at 100 frames per second. Tracking software was utilized to extract the salient kinematics of the birds manoeuvring and interacting in their natural environment. The recording were conducted on a colony of approximately 50 birds at the North Carolina Highway 751 bridge over Jordan Lake, Chatham County, NC, USA, 35° 49′ 42′′ N and 78° 57′ 51′′ W (latitude, longitude). The dataset analysed here comprises the 3D X-, Y-, and Z-position, the velocity and acceleration vectors obtained using first and second time derivatives. The length of the datasets varies between 0.72 to 4.71 seconds and a smoothing spline method was applied to birds position with known uncertainty for each position parametrized from the stereo retro-projection error in the cameras, see^61^ for further methodological details. The datasets are also publicly available in the website http://biomech.web.unc.edu/datasets/.

We investigate leadership in pairs of birds using only transfer entropy. In fact, given the short duration of the recordings resulting in few number of spikes present in the dataset, directed event synchronization which is based on the synchronicity between spikes could not be implemented. To apprehend the full 3D dynamics of the natural flight dataset which includes widely varying speeds, turns, and roll manoeuvres, we used the acceleration time series as our variable of interest to implement the method instead of the turnrate, which is more appropriate in cases of constant speed and 2D motion. We start our analysis by implementing our method over different time window lengths and also over the entire length of the dataset in order to compare our results to traditional approaches^43,70^ which consider leadership as constant over the entire length of the dataset.

Prior to implement the method on tandem flights, the threshold value to differentiate between true and false links is computed using surrogate data obtained by randomly pairing the first trajectory in a given trial to the second trajectory of a different trial. Note that in this dataset the first bird is always ahead of the second bird along the shared flight trajectory and was termed the leader in the original analysis^61^. Pairwise interactions ${A}_{({\rm{TE}})}^{(ij)}$, for *i* ≠ *j* = 1, 2 were computed on this new dataset consisting of virtually interacting birds and we retained the upper confidence interval bound of a t-test at 5% confidence level. We repeat the same procedure 30 times and compute the average value of the upper confidence interval bound (0.1190 ± 0.0217) which is taken as threshold ${\bar{A}}_{{\rm{TE}}}$. In the calculations, transfer entropy is evaluated using 10 bins for the tandem flight dataset. To validate the leader identity in pair of tandem flight using traditional methods with transfer entropy, we performed a paired t-test which returns a *t*-value (*t*(.)) with degree of freedom (.) and a *p*-value. The upper bound confidence interval is utilized to differentiate between leaders and followers in each trial.

## Results

### Detecting intermittent leadership in the Vicsek model

#### Threshold value and time window length

Using a sample dataset of pairs of non-interacting Vicsek particles, the upper bound confidence interval of the mean value of ${A}_{(\cdot )}^{(ij)},i\ne j=\mathrm{1,}\,2$ is computed as ${\bar{A}}_{{\rm{TE}}}=0.0040$ for transfer entropy and ${\bar{A}}_{{\rm{ES}}}=0.0091$ for directed event synchronization. These values are retained as the threshold value for the Vicsek datasets.

The predicted switching time returned by successively increasing the time window length (*win*) from 100 to 2,000 time steps are presented in Supplementary Information Table S3. In the Table, we observe that for both transfer entropy and event synchronization, the algorithm parametrized by time intervals from 800 to 1,000 time steps tends to present a higher success rate and accurate values of the switching time. For other time windows, the algorithm exhibits low success rate and large variability of the switching time. Thus, for the Vicsek model, we retain in the analysis an investigation time window of length 1,000 time steps.

#### Performance of the method in *in-silico* experiments

**Figure 4:**
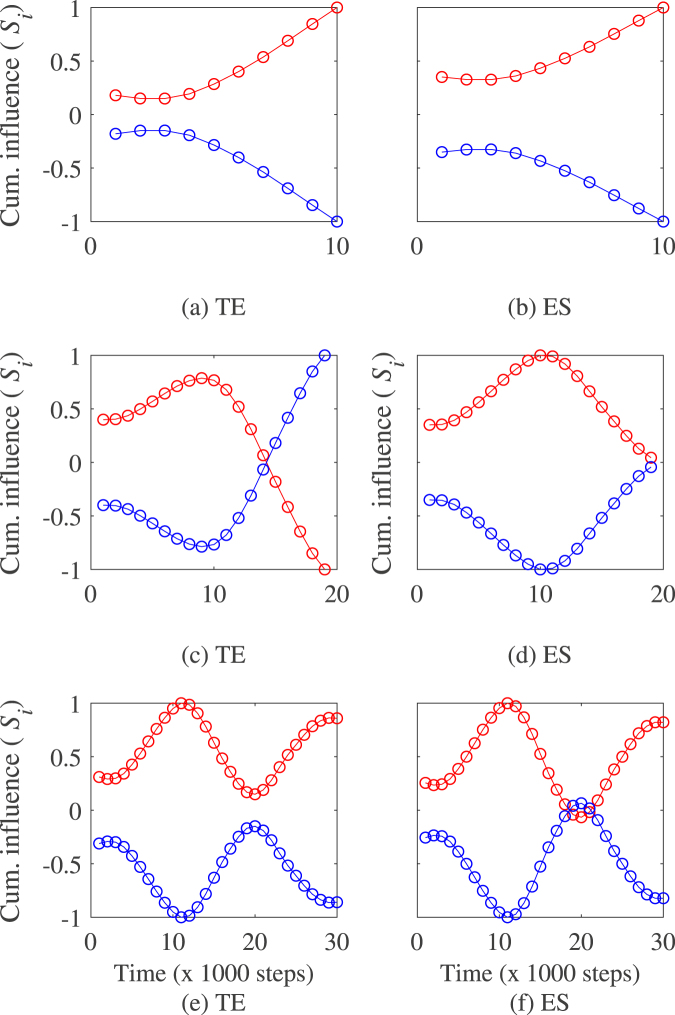
Illustration of switching leadership detection on simulated Vicsek particles with (**a**,**b**) constant leadership, (**c**,**d**) a single switching leadership event, (**e**,**f**) two leadership switching events. Left plot indicates the influence of individual 1 on individual 2 and right plot is the influence of 2 on 1. Note that the values of the cumulative influence (*S*_i_) are normalized between −1 and 1. Model parameters for the simulations are *N* = 2, *K* = 2, *v* = 0.05, *η* = 0.2, and the switching events are set at 10,000 and 20,000 time steps after discarding the first 10,000 steps.

Fig. 4(a,b) illustrates the method on a pair of Vicsek particles with a single and constant leader. In the figure, the cumulative influence of particle 1 on particle 2 is monotonic increasing indicating that our method is capable of predicting that leadership (resp. follower role) is sustained for particles 1 (resp. particle 2) during the entire length of the experiments. Table 1 presents the accuracy of the method measured by the ratios of true positive (TPR) and false positive estimated (FPR). Results in the table show that the method provides with a good accuracy rate that in average, particle 1 sustains leadership all along the observation period TPR = 0.97 and FPR = 0.03 for transfer entropy, and TPR = 0.93 and FPR = 0.10 for directed event synchronization respectively.

**Table 1:** Performance of the method and estimated leadership switching time in the Vicsek model.

Measure	Switching events	Sensitivity (TPR)	Specificity (FPR)	Time (s)	std (s)
TE	0	0.97	0.03	—	—
	1 of 1	1	0	10,200	847
	1 of 2	1	0	10,167	531
	2 of 2	1	0	18,800	407
ES	0	0.93	0.10	—	—
	1 of 1	1	0	10,167	379
	1 of 2	1	0	10,000	455
	2 of 2	1	0	18,866	507

Figure 4(c,d) illustrates the method for a pair of Vicsek particles with leadership switching from individual 1 to 2 at a predefined time step. Results in Table 1 show that the method predicts change of leadership with a good accuracy rate estimated at TPR = 1 and FPR = 0 for both transfer entropy and directed event synchronization respectively. In addition, the method provides a good detection of the leadership switching time estimated at 10,200 ± 847 for transfer entropy and 10,167 ± 379 for directed event synchronization respectively.

Figure 4(e,f) provides an illustration of the method with two leadership switching events. Results in Table 1 show that the intermittent causal relationship detection algorithm predicts these changes of leadership with a very good accuracy rate (TPR = 1 and FPR = 0) for transfer entropy and directed event synchronization. In addition, the method provides a good prediction of the switching time for the first change of leadership while for the second one, the predicted time is about 1000 steps different from their predetermined value for both transfer entropy and directed event synchronization. Such a difference is inherent to the interpolation procedure to estimate the best differentiable function approximating the cumulative influence function which might result in a predicted time slightly different from its true value.

#### Resilience of the method to noise perturbation

**Figure 5:**
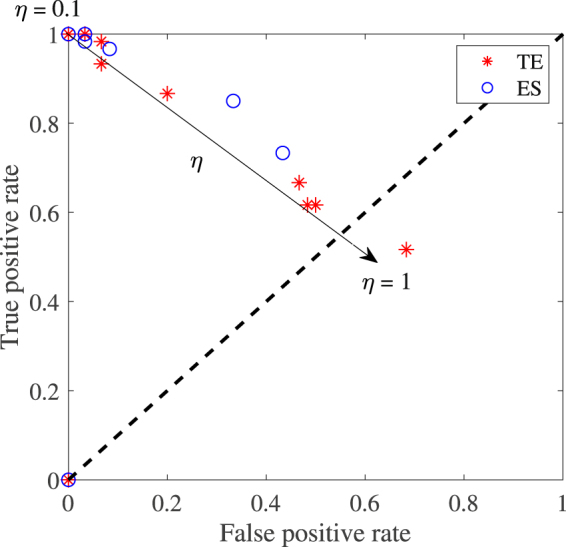
Performance of the method for transfer entropy (red stars) and directed event synchronization (blue circles). This ROC is parametrized here by noise intensities from 0 to 1 in 0.1 increments, that is *η* = 0.1, 0.2, …, 1 (black arrow). The threshold utilized is identical for all point (${\bar{A}}_{{\rm{TE}}}=0.0040$ and ${\bar{A}}_{{\rm{ES}}}=0.0091$). Note that the point (0, 0) correspond to noise level *η* = 0 and other model parameters retained in the simulations are *N* = 2, *K* = 2, *v* = 0.05.

Figure 5 depicts the performance of the method for increasing noise level. Results show that as noise is progressively increased (arrow direction) in the system, the performance of our method evaluated by the receiver operating characteristic (ROC) varies from the top left corner for small noise values to the diagonal dashed line for large noise intensities above 0.5. This trend indicates that, for small noise intensities, the method correctly classifies leaders and followers and as noise is increased, the method can no longer clearly differentiate between leaders and followers. We present also in Table 2 the time leadership is predicted to switch from particle 1 to 2. In the Table, we observed also that as noise is injected into the system, the error in predicting the switching time considerably increases as attested by their estimated standard deviation.

**Table 2:** Predicted leadership switching time (mean and standard deviation) between particle 1 and 2 as a function of the noise intensity.

	*η*	0.1	0.2	0.3	0.4	0.5	0.7	0.9	1
TE	mean	9,462	9,607	9,828	10,069	9,704	9,958	10,632	9,250
	std	1,881	685	602	1,534	1,171	2,274	3,562	2,896
ES	mean	9,767	9,633	9,931	9,724	9,862	9,680	10,375	10,214
	std	430	669	530	797	789	1,069	3,202	3,577

Note that in the total absence of noise, the particles reaches consensus at steady state and the algorithm cannot differentiate leaders from followers. In such a case, one needs to introduce of a slight perturbation in the system or to consider an appropriate time delay to capture synchronicity and eventually a change of leadership. For a system with more than two followers, indirect interactions between followers might be observed resulting in delays between, for example, a turning manoeuvre initiated by the leader and the response of a distant follower. However, since the influence of group leaders is constant over time as compared to a follower transmitting the information to another group member, one should expect group leaders to exert a much stronger influence on the rest of the group in the long run^43^.

#### Multiple switching at different frequencies

**Figure 6:**
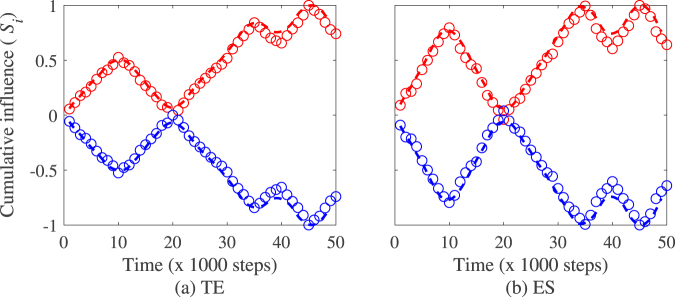
Time trace of the cumulative influence index of particle 1 on particle 2 (red) and particle 2 on particle 1 (blue) in an experiment where five switching events are simulated including 2 occurring after 10,000 time-steps, one after 15,000 time-steps, and 2 occurring after 5,000 time-steps. Note that the values of the cumulative influence (*S*_i_) are normalized between −1 and 1. Model parameters retained in the simulations are *N* = 2, *K* = 2, *v* = 0.05, and *η* = 0.2.

Figure 6 depicts the time trace of the cumulative influence index of each particle on the group as they transition either from leader to follower or from follower to leader over five successive switching events at different frequencies. The values in Table 3 show that the method provides a good prediction time of the change of leadership at the three predefined frequencies with a performance estimated at TPR = 0.95 and FPR = 0.04 for transfer entropy and TPR = 1.0 and FPR = 0.0 directed event synchronization respectively.

**Table 3:** Predicted leadership switching time in the Vicsek model for multiple events at different frequencies.

Events	Frequency	TE		ES	
		mean	std	mean	std
1 of 5	10,000	10,033	663	10,000	—
2 of 5	10,000	20,233	1,212	19,967	181
3 of 5	15,000	34,633	920	35,000	—
4 of 5	5,000	39,700	1,013	40,000	—
5 of 5	5,000	45,167	693	45,033	181

#### Group of more than two

**Figure 7:**
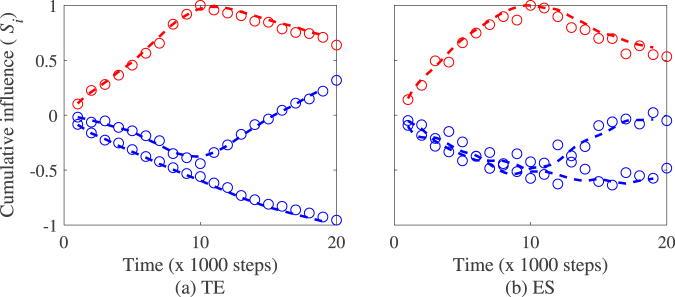
Time trace of the leadership index for particle 1 (red), particle 2 (blue reaching a minimum), and particle 3 (blue decreasing) in an experiment where a single switching event is simulated and each particle is set to interact in average with a single one. Note that the values of the cumulative influence (*S*_i_) are normalized between −1 and 1. Model parameters retained in the simulations are *N* = 3, *K* = 1, *v* = 0.05, and *η* = 0.2.

Figure 7 presents the behaviour of the leadership index in the experiment simulating three particles with in average at most a single link between particles. The average leadership switching time returned by the method is 9,950 ± 153 time-steps. The prediction of the switching leadership TPR and FPR estimated at 1 and 0 for TE and 0.97 and 0.07 for ES. Overall the method is capable to predict change of leadership event in the case the leader might not directly influences a specific individual.

**Figure 8:**
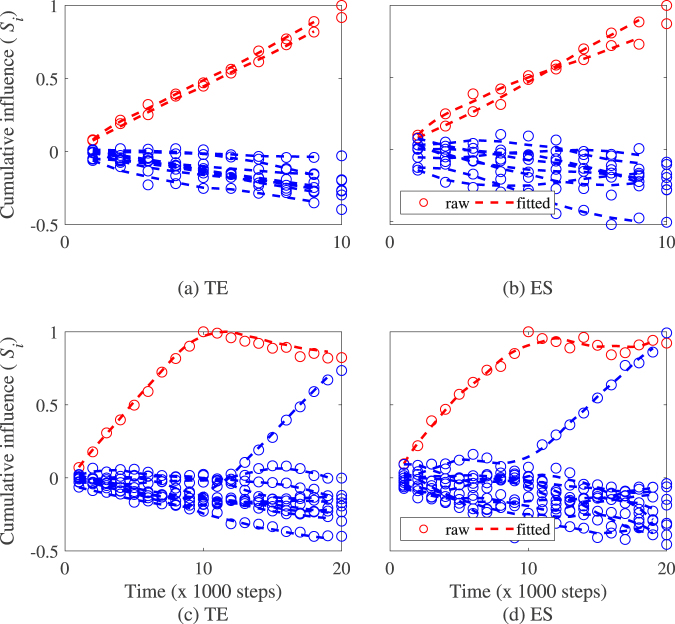
Illustration of the method on Vicsek model with N = 10 particles with no switching event (**a**) and (**b**) and a single switching leadership event (**c**) and (**d**). In (**a**) and (**b**), the time trace of the cumulative influence index for leaders is increasing while decreasing for the followers. In (**c**) and (**d**), the time trace of the leadership index of the particles switching from either leader to follower (red) or follower to leader (blue transition) showed a significant change in their monotonicity as time evolved. Note that the values of the cumulative influence (*S*_i_) are normalized between −1 and 1. Model parameter are *N* = 10, *K* = 1, *v* = 0.05, and *η* = 0.2. Note that *K* = 1 indicates that in average each particle interacts with a single neighbour.

Figure 8(a,b) illustrates the behaviour of the cumulative influence index for each particle for both transfer entropy and directed event synchronization with leaders cumulative influence index increasing while followers index decreases. This behaviour is observed for all 30 sample datasets simulated. Figure 8(c,d) depicts the time trace of the leadership index for a group of *N* = 10 particles including a switching event after 10,000 time steps. In the Figure, followers leadership index is monotonic decreasing while it reaches a maximum (resp. minimum) for individuals whose state change from leader to follower (resp. follower to leader). The method predictive performance given by the TPR and the FPR is 0.99 and 0.1 respectively for transfer entropy with a predicted switching time of 10,000 ± 68-time steps. For even synchronization, the TPR and the FPR are 0.98 and 0.13 respectively with the switching time predicted at 9,870 ± 778-time steps.

### Detecting switching leadership in fish shoaling

#### Threshold value and time window length

The upper bound of the confidence intervals of the mean of ${A}_{(\cdot )}^{(ij)},i,j=\mathrm{1,}\,2$ determined using a t-test at 5% confidence level is ${\bar{A}}_{{\rm{TE}}}=0.0113$ for transfer entropy and ${\bar{A}}_{{\rm{ES}}}=0.0165$ for directed event synchronization. These values are utilized as thresholds for pairwise interaction in the rest of the analysis.

The results in Supplementary Information Table S4 obtained by varying the time window length to parametrize the method show that the interval of length between 4 to 6 seconds provides good estimates of the predefined switching time for transfer entropy with in addition a high success rate and a low standard error. For directed event synchronization, only time interval length between 4 and 5 seconds tend to provide acceptable results. Finally, we set the detection time window length to 5 seconds in the analysis of fish shoaling datasets.

#### Performance of the method in *in-silico* experiments

**Figure 9:**
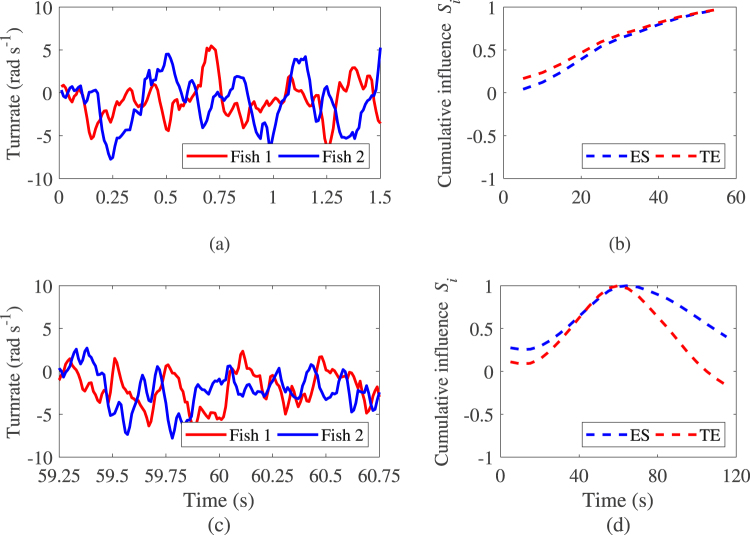
Illustration of the time trace of a sample fish turnrate and of the cumulative influence of fish 1 in the absence and in the presence of a switching leadership event. The figure depicts the evolution of the turnrate and the resulting effect on the cumulative influence index as time evolves. Turnrate time trace is limited to facilitate the visualization of the times series. Note that the values of the cumulative influence *S*_*i*_ are normalized between −1 and 1. Model parameters for the simulations are summarized in Supplementary Information Table S2.

In Fig. 9(a,b), we illustrate the time trace of the turnrate and of the cumulative influence in case of constant leadership and when a change of leadership between fish 1 and 2 is observed. Figure 9(a) is a sample time trace of the turnrate for pair of fish including a constant leader. In this figure, one can see that spikes in the time series of fish 1 always precede those in the times series of fish 2 resulting in Fig. 9(b) in a monotonic increasing cumulative influence of fish 1 on 2. In case of switching leadership with time trace of the turnrate illustrated in Fig. 9(c), one can observe a change of trend in the time series near t = 60 seconds where spikes in time series of fish 2 begins to precede those in the time series of fish 1. This trend results in the cumulative influence of fish 1 on fish 2 reaching a maximum value before decreasing as shown in Fig. 9(d).

The performance of the algorithm is presented in Table 4 with the true and false positive rates, and the predicted leadership switching time over the 30 sample datasets. The method implemented on both transfer entropy and directed event synchronization tends to provide good predictions of the predefined switching leadership event with true positive rate values close to 1 and false positive rate values close to 0. In addition, the error in predicting the leadership switching instants is small and the estimated times fall within a good confidence interval of their predefined values.

**Table 4:** Performance of the method and estimated time of leadership switching in the fish shoaling.

Measure	Switching events	Sensitivity (TPR)	Specificity (FPR)	Time (s)	se (s)
TE	0	1	0.02	—	—
	1 of 1	0.98	0.05	59.1	1.5
	1 of 2	1	0	60.3	2.2
	2 of 2	1	0	120.8	2.6
ES	0	1	0	—	—
	1 of 1	0.98	0.03	62.1	2.1
	1 of 2	1	0	63.0	2.0
	2 of 2	1	0	117.7	2.8

### Detecting switching leadership in tandem bird flight

#### Threshold value and time window length

The threshold value to differentiate between true and false links is estimated as ${\bar{A}}_{{\rm{TE}}}=0.1190$. To determine an appropriate window size to investigate for switching leadership in the tandem bird flight, we construct a ground truth dataset exhibiting switching leadership. The procedure consists of taking a genuine bird dataset where constant leadership is observed from bird 1 to bird 2 independently of the window size and merging it to its copy where the order of the birds has been flipped to invert the direction of the information flow. A small moving average is applied to smooth the transition between the two merged datasets. Supplementary Information Figure S1 illustrates the time trace of the cumulative influence index as the switching leadership detection window size is varied from 0.15 to 0.40 s. A predictive performance comparison shows that the window size (*win* = 0.25 *s*) provides the best leadership switching detection in tandem bird flight.

**Figure 10:**
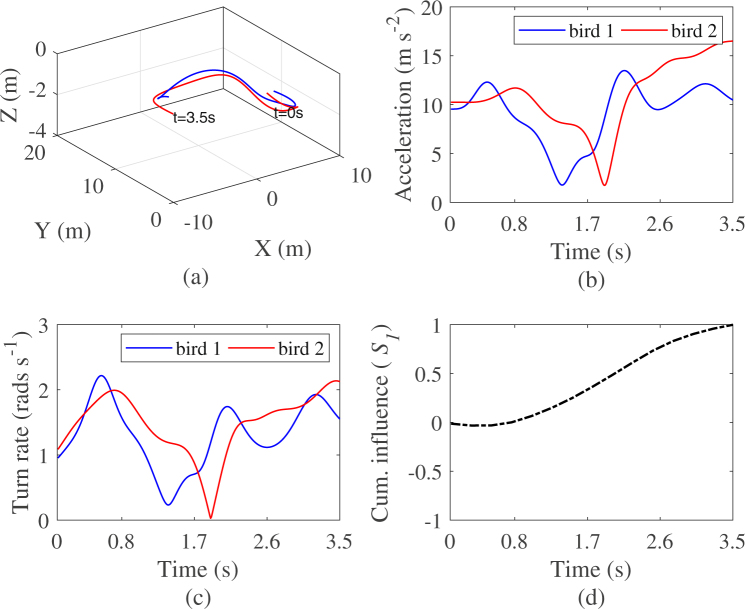
Time trace of the kinematics of a sample tandem flight with (**a**) birds 3D position, (**b**) acceleration, (**c**) absolute turn rate^61^, and (**d**) time evolution of the cumulative influence of bird 1 on 2 resulting in a consistent leadership over time. Note that the values of the cumulative influence in (d), are normalized between −1 and 1.

**Figure 11:**
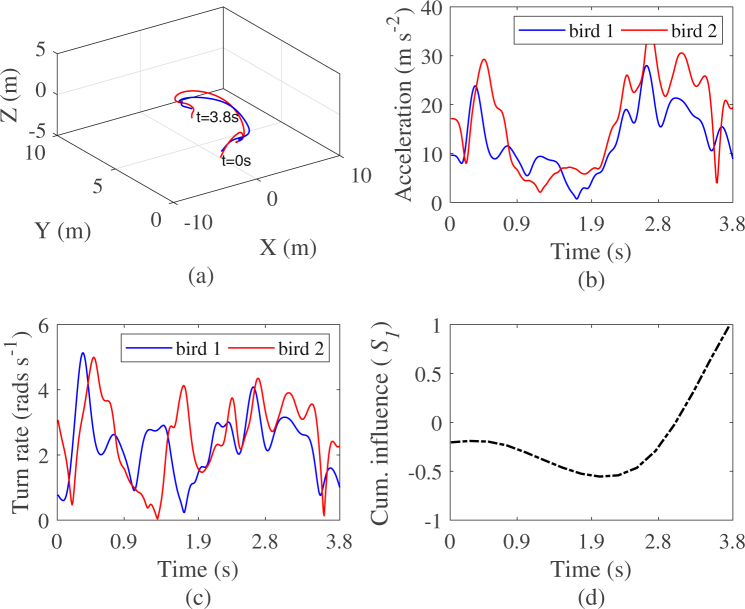
Kinematics of a sample tandem flight with (**a**) birds 3D position, (**b**) acceleration, (**c**) turn rate, and (**d**) time trace of the cumulative information flow from bird 1 to bird 2. Leadership switches from bird 2 to bird 1 about 2.25 s after the beginning of the video recordings. Note that the values of the cumulative influence in (d), are normalized between −1 and 1.

To implement the algorithm, we discard any local extrema that is not preceded and then followed by at least two consecutive increasing or decreasing values of the cumulative influence function. We also do not consider any local extrema that fall close to the beginning or at the end of the observations period. These restrictions are intended to avoid false positives in detecting the local maximum and minimum. Note also that in the absence of a well known exemplary trial of cliff swallow tandem flight exhibiting change of leadership, we additionally verify the accuracy of the predicted switching time by comparing the predicted values in Supplementary Information Table S5 to the time trace of the trajectories, the turnrate, and the acceleration (see Figs 10 and 11) in order to identify any specific change of values of these kinematics at the given instant that might explain such change of leadership. The videos recordings of the flights are also visualized to validate our findings (see Supplementary Information Videos 1 and 2).

#### Comparison with traditional approaches

We recall that in traditional approaches, leadership is hypothesized to be consistent over the duration of the observations and the measures of causality are implemented on the entire length of the data. Results are summarized in Table 5 for trials with change of leadership during the tandem flight. In Table 5, the first column is a number identifying one of the 39 sample datasets retained for the analysis, the second column presents the net transfer of information flow between bird 1 and 2 over the entire length of the dataset, the third column indicate the individual identified as group leader through this traditional approach, the fourth column are the estimated time instant where leadership is idenfied to change from one individual to another one, and the last column indicates from what individual to other one leadership is observed to switch. In Supplementary Information Table S5, we present also salient kinematics of bird locomotion in 3D including speed, acceleration, and turning rate^61^.

**Table 5:** Prediction of leadership in the tandem flight using transfer entropy over the entire length of the dataset and by implementing the intermittent leadership detection algorithm over time window length of 0.25 second.

Data	netTE^1→2^	Leading bird	time(s)	Switching leadership (bird *i* → *j*)
4	0.011	1	3.00	2 → 1
7	0.039	1	2.25	2 → 1
8	−0.007	—	0.75	2 → 1
10	0.011	1	0.75	2 → 1
11	0.016	1	3.50	1 → 2
17	0.000	—	3.00	1 → 2
18	−0.005	—	0.75	2 → 1
21	0.029	1	3.75	2 → 1
24	−0.002	—	0.75	2 → 1
27	0.018	1	0.75	1 → 2
28	0.023	1	0.75	2 → 1
30	0.047	1	0.75	2 → 1
34	0.000	—	3.00	1 → 2
35	0.034	1	0.75	1 → 2
37	0.036	1	0.75	1 → 2
39	0.042	1	0.75	2 → 1
mean	0.018*	—	1.63	—
std	0.005	—	0.3	—

Net transfer entropy netTE^1→2^ = TE^1→2^ − TE^2→1^ is utilized to assess the directionality of the information flow with * indicating significance using a two-tailed t-test at 5% confidence level. Third column indicates the predicted leader using traditional consistent leadership approaches in individual datasets based on the confidence interval of a t-test evaluating if bird 1 is the leader ${(\mathrm{netTE}}^{1\to 2} > 0)$, or bird 2 is the leader (netTE^1→2^ < 0). The symbol − depicts either not significantly detected as leader (column 3) or no switching event detected. Only trials with change of leadership are shown here. See Supplementary Information for all datasets in Table S5.

In Supplementary Information Table S5, assuming consistent leadership, transfer entropy applied on the entire dataset predicts that on average, more information flow is transfered from bird 1 to bird 2, meaning that on average bird 2’s flight behaviour is in reaction to bird 1’s behaviour. This result is validated by a paired t-test (*t*(38) = 5.23, *p* < 0.01). We use also the confidence interval bounds of a t-test to compute a threshold to assess group leader in the individual datasets. Results in Supplementary Information Table S5 show that bird 1 influences the behaviour of bird 2 in 22 trials while in 2 trials, bird 2 is identified as the leader. For the other 15 trials, the confidence interval of the t-test failed to reveal which bird is definitively leading the other. Compared to the above traditional analysis of cause effect relationship, the novel method to detect intermittent causality suggests in column 4 and 5 of Table 5 that leadership might not be constant over the observation length for all trials. In 16 of the 39 trials, the leader-follower relationship between bird 1 and 2 might change within the observation period.

In Fig. 10(a–d), we illustrate an exemple scenario of consistent leadership where the cumulative influence of bird 1 on 2 evaluated by our method is monotonic increasing, indicating that individual 1 is constantly influencing the flight behaviour of individual 2 over the entire length of the observation (Table 5 data 2). Such consistent leadership might not always hold over the entire length of the time series as illustrated in the second example in Fig. 11(a–d), where the cumulative influence function initially decreases, indicating more information flow from bird 2 to bird 1 before reaching a minimum where it starts to increase indicating a change into the directionality of the information flow which is now observed to be directed from bird 1 to bird 2 (Table 5 data 7). In case of constant leadership along the observations, one can observe in Fig. 10(a–c) that the time trace of the position, the acceleration, and the turn rate evolve more smoothly as compared to Fig. 11(a–c), where in case of switching leadership, the values of the same kinematics tend to depict sharp turning manoeuvres and large fluctuation of the acceleration and the turn rate (see Supplementary Information Videos 1 and 2). Note that for the first dataset, although the leadership role is consistent through time, transfer entropy applied to the entire dataset does not assign bird 1 the leader role with *p* < 0.05 although net transfer entropy is positive. In the second case, transfer entropy applied over the entire length of the dataset does assign bird 1 the leader role with *p* < 0.05. Hence, in the first example, while traditional method failed to significantly identify group leader, the novel method tend to indicate that more information flow from bird 1 to bird 2. In the second example, while traditional methods failed to reveal the dynamics of the causal influence, the novel method clearly indicates change in leadership.

## Discussion

Understanding the mechanisms at the base of leader follower relationship in natural systems is fundamental to explain the interaction between individuals in biological groups and for potential applications in science and engineering. In the study of such causal relationship, current methods do not provide the possibility to dissect potential dynamics that might exist in group leadership. In this work, we propose a method to investigate intermittent change in causal relationship in coupled networked dynamical systems. The method utilizes transfer entropy or directed event synchronization to compute the cumulative influence of individuals on the rest of the group over successive time windows. A significant decrease in slope or an extrema detected into the cumulative influence function as local maximum indicates a change from leader to follower while a significant increase in slope or a local minimum depicts a switching of influence from follower to leader.

Our method is initially implemented on synthetic datasets where the leader-follower relationship can be controlled and a given uncertainty can be introduced. First, the method is tested on Vicsek self-propel particles model^51^ which offers the possibility to test the resilience of our method to increasing noise. In this framework, we have shown using indicators of performance such as True positive rate (TPR) and the False positive rate (FPR), that, upon selecting appropriate investigation window length to segment data, the information-based statistics can predict changes in leadership for pairs at identical or different frequencies and for multiple individuals. We have also demonstrated that our method clearly differentiates change of causal influence up to a noise level above 0.5 where its performance decreases drastically. This trend is similar to the order parameter in the Vicsek model where for increasing noise level, a phase transition from order to complete disorder is observed^67^.

The method is also tested on an authentic dataset generated by a data-driven model whose parameters are calibrated against experimental data to reproduce realistic fish social behaviour^52,53,69^. Unlike the Vicsek model where leaders have a constant heading angle, in the fish shoaling model, the leader’s heading angle is unpredictable and controlled by a stochastic process. Through a social response function, the follower are coupled to the leader and the rest of group where they either align their heading direction, come closer to each other, or interact with their environment such as walls and obstacles. In this highly stochastic synthetic datasets of fish shoaling, we show that, choosing appropriate window size, for a pair of individuals exhibiting change in leadership, the information-based statistics can detect it.

Finally, we tested our method on datasets depicting the interaction of pairs cliff swallows in tandem flight and recorded in the nature. These datasets offer the possibility to test our algorithm on real world datasets of animals interacting freely in their natural environment as opposed to laboratory settings where animal behaviour might be confined by the dimensions of the setup and other factors such as social dominance. An implementation of a traditional approach to detect causal interaction indicates that in most trials, the bird flying in front (and identified as bird 1) might influence the flight behaviour of the bird flying just behind (and identified as bird 2). Our algorithm suggests instead that, this causal relationship varies over time between individuals in accordance with recent literature on animal behaviour^44,45^, and that bird 2 may herd bird 1 instead of following it as the interaction develops. Such change of leadership during the observations whereas an individual identified as leader at a given time instant might switch to a follower role at another time of observation has been demonstrated in^45^ using a network approach based on functional mapping between sensory input and behavioural output. These findings suggest that prior assumptions of consistent leadership might hinder understanding natural group dynamics in animals.

Change in leadership as suggested by our method in cliff swallow is characterized by sharp turns observed in their trajectories resulting in high peak values in their acceleration and turn rate dynamics. These manoeuvres depict a non-verbal communication by the bird initiating the manoeuvres and are likely intended to influence the flight behaviour of the other bird. These manoeuvres are more energetically expensive than smoother pursuits with smaller magnitude accelerations and likely indicate a more competitive contest between the two birds. Such actions may also be related to boldness which is a risk-taking behaviour observed in several other animal species with the purpose to act on the behaviour of a conspecific^44^.

Cliff swallows (*Petrochelidon pyrrhonota*) in particular are well known for their highly social behaviour that includes but is not limited to foraging and nest-building^71^. They are also known for their highly manoeuvrable flight, which enables them to hunt flying insects^72^ and importantly, these birds are reported to be conspecific nest parasites^73^. As reported in^61^, the tandem flights analysed here consist of competitive interactions with an intruder approaching a guarded nest. Such behaviour is often observed during their nesting period near the nest colony^61^. It is thus, tenable to hypothesize that the intensive flight manoeuvres of these contests might include intermittent changes in leadership, where the rear bird (bird 2) might be termed as chasing (i.e. bird 1 is the leader) or herding (i.e. bird 2 is the leader) while defending the nest.

The method proposed in this manuscript is intended to enrich the methodological toolbox to investigate leader-follower relationship in coupled networked dynamical systems including but not limited to animal groups and swarm robotics. It complements prior investigation tools by offering a mean to unravel intermittent and switching causality in coupled networked dynamical systems. Limitation of current approach includes systems achieving consensus in the absence of noise or or evolving at very low noise level. For such systems, a slight perturbation of the system dynamics or the use of appropriate time delay is required to allow for leadership detection. Transfer entropy and directed event synchronization retained here to evaluate causal relationship need to be parametrized with appropriate time delay. For large network size, the method requires extensive computational resources to evaluate pairwise interactions and analysing the monotonicity of individual’s cumulative influence index. The computational time might be addressed by parallelising or optimising the procedure and future works might extend the method to dissect intermittent leadership in other animal species or in swarm robotics.

## Electronic supplementary material

Supplementary Information

Supplementary Video 1

Supplementary Video 2
