# Supplementary material for: Detecting intermittent switching leadership in coupled dynamical systems
Source: Sci Rep. 2018 Jul 9;8:10338. doi: 10.1038/s41598-018-28285-1 (PMC6037816; doi:10.1038/s41598-018-28285-1)
Supplement: Supplementary file 1 — Supplementary Information [file 41598_2018_28285_MOESM1_ESM.pdf]

Detecting intermittent switching leadership in coupled dynamical systems

Violet Mwaffo*[1], Jishnu Keshavan[1], Tyson Hedrick[2], and Sean Humbert[1]

[1]Department of Mechanical Engineering, University of Colorado, Boulder, CO, USA

[2]Department of Biology, University of North Carolina at Chapel Hill, Chapel Hill, NC, USA

Supplementary information

Supplementary Figure S1

Effect of various window size ($win = 0.15 : 0.05 : 0.40s$) on the cumulative influence index of bird 1 on bird 2 computed on a sample bird tandem flight dataset (left column) and a reconstructed ground truth dataset exhibiting switching leadership (right column). The windows size of 0.25 and 0.30s tend to provide good prediction of the leadership switching time. However, only the window size of 0.25s provides a unique and correct switching time while the window size of 0.30s tends to display a plateau with a maximum over a wide interval of time.

*Author for correspondence (violet.mwaffo@colorado.edu)

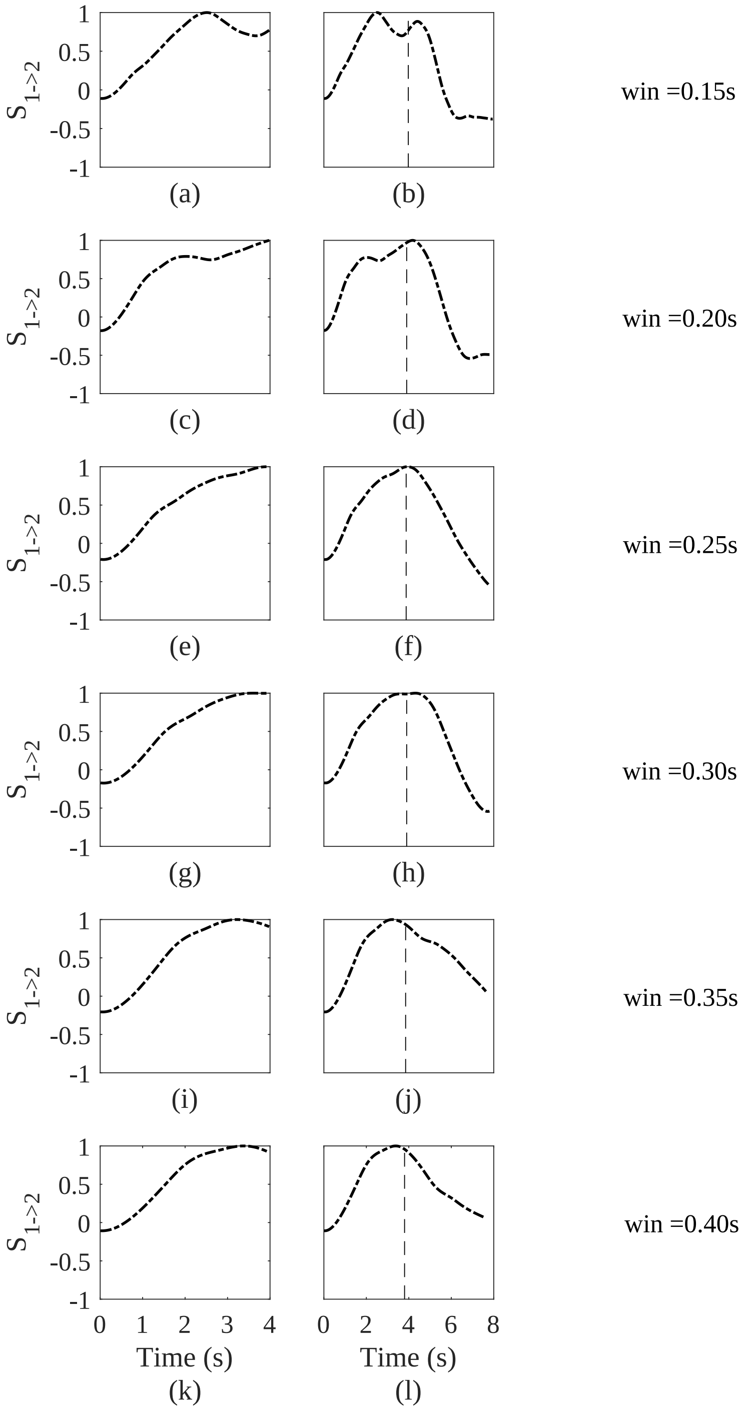
S1->2
win =0.15s
win =0.20s
win =0.25s
win =0.30s
win =0.35s
win =0.40s
Time (s)
(a)
(b)
(c)
(d)
(e)
(f)
(g)
(h)
(i)
(j)
(k)
(l)

Supplementary Table S1

Step	Procedure	Output
1.	Select a measure of causality denoted (.) e.g. Transfer entropy or event synchronization	e.g. transfer entropy, causation entropy, event synchronization, spike synchronization
2.	Generate a sample shuffled dataset or a sample surrogate dataset where no interaction exists between individuals	Surrogate dataset with no coupling between time series
3.	Using the above dataset, compute for each trial the quantities $A^{ij}_{(\cdot)}, i, j = 1, \ldots, N$ and use it to determine a threshold value $\bar{A}_{(\cdot)}$ as the upper bound confidence interval of a t-test for example	Threshold value $\bar{A}_{(\cdot)}$ to exclude false interactions
4.	Use a ground truth dataset exhibiting change in leadership at a known point in time to determine an optimal windows interval *win* to detect switching leadership. The dataset can be generated using a data-driven model reproducing similar behaviour or by constructing one exhibiting switching leadership.<br>For the later, consider a segment of data exhibiting a one-way interaction independently of the segmentation considered. Make a copy of the dataset, flip the order of individuals to revert the direction of interaction and merge it to the original dataset to obtain a new dataset exhibiting switching at mid-length.	Optimal window interval *win* for leadership switching detection

5.	Compute the cumulative influence index $S^{(i)}_{(\cdot)}(t_{seg}) = \sum_{t_{win}=1}^{t_{seg}} \Delta S^{(i)}_{(\cdot)}(t_{win}), i = 1, \ldots, N$ by evaluating the network divergence $\Delta S^{(i)}_{(\cdot)}(t_{win})$ over successive time window. The returned time series need to be smoothed and an interpolation procedure need to be applied to determine a differentiable function $\hat{S}^{(i)}_{(\cdot)}(t)$ allowing to easily study its monotonicity	cumulative influence index $S^{(i)}_{(\cdot)}(t_{seg})$, and $\hat{S}^{(i)}_{(\cdot)}(t)$, for $i = 1, \ldots, N$
6.	Determine the extrema of the cumulative influence function $\hat{S}^{(i)}_{(\cdot)}(t), i = 1, \ldots, N$ (a maximum indicates a switching from leader to follower, a minimum indicates a switching from follower to leader, an increasing function indicates a constant leader, and a decreasing function indicates a constant follower).	Extrema indicating the number of switching events and the corresponding switching time instant
7.	In case of more than a switching event, correct the value of the switching time by considering instead the time instant corresponding to the nearest peak in the raw values of the index to the peak observed in the smoothed values of the index.<br>Note that the interpolated index returns the number of switching events and the switching times. However, due to numerical interpolation, if more than a switching event is observed, it might shift the location of the extrema undermining the true value of the switching time.	Corrected values of the leadership switching time
8.	A leadership order can be established by implementing the method several times by discarding the most or the less influential individuals till the order of leadership is clearly established. Note also that in case of more than two individuals, another threshold value can be set to reduce the computational time by quickly differentiating between potential leaders and followers. In this case, the monotonicity of the cumulative influence index be restricted to potential leaders.	Leadership order

Supplementary Table S2

Model parameters retained to replicate fish shoaling in the simulations.

Parameter	Description	Value
$\alpha^{(i)}$	Relaxation rate	1/0.024 (m^{-1})
$\sigma^{(i)}$	Turn rate variability	28.9 ($m^{-1}\,rads^{-1/2}$)
$v^{(i)}$	Speed	0.564 ($m\ s^{-1}$)
$k_W^{(i)}$	Wall avoidance control gain	4.7 (s^{-1})
$k_v^{(i)}$	Weight of alignment	27 (m^{-1})
$k_p^{(i)}$	Weight of positional interaction	0.41 ($m^{-1}\,s^{-1}$)
Δt	Time step	0.01 s

Parameters are calibrated on experimental data of fish shoaling in a large water tank.

Supplementary Table S3

Sensitivity analysis of the estimated switching time as a function of the time window length (win) used to parametrize the method in the Vicsek model.

	TE			ES		
win (time steps)	mean	se	# success	mean	se	#success
100	9175.0	47.9	4	9171.4	49.6	14
200	9415.4	47.8	13	9352.0	52.0	25
300	9478.6	127.6	14	9534.6	75.0	26
400	9765.2	139.8	23	9666.7	108.9	27
500	9640.0	183.8	25	9862.1	69.7	29
600	9200.0	327.7	19	9485.7	276.6	21
700	9192.0	146.2	25	9616.7	220.3	30
800	9542.9	123.8	28	9427.6	101.1	29
900	10172.4	122.5	29	10190.0	134.3	30
1000	10900.0	236.6	30	11100.0	73.5	30
1100	11925.0	192.9	28	12236.7	64.2	30
1200	12892.3	178.3	26	13000.0	132.9	30
1300	13187.0	529.1	23	13240.9	396.4	22
1400	12353.8	1010.2	13	12800.0	760.6	14
1500	7250.0	461.0	10	8500.0	723.0	12
1600	7320.0	320.0	15	7960.0	640.0	10
1700	8350.0	580.7	6	7742.9	165.0	14
1800	8300.0	300.0	6	8000.0	0.0	7
1900	8880.0	380.0	5	8500.0	0.0	7
2000	9000.0	0.0	4	9000.0	0.0	1

Note that success corresponds to the number of cases the method return a value.

Supplementary Table S4

Sensitivity analysis of the predicted switching time as a function of the time window (*win*) length used to parametrize the method in the fish shoal model.

	TE			ES		
win (s)	mean(s)	se(s)	# success	mean(s)	se(s)	#success
1	-	-	0	-	-	0
2	75.0	13.0	2	71.3	1.3	3
3	63.0	2.0	13	69.9	2.7	14
4	62.0	2.5	26	63.8	3.3	22
5	60.0	1.4	26	60.7	2.0	22
6	67.9	1.2	28	67.0	2.1	25
7	75.1	1.3	22	76.6	1.5	19
8	78.4	3.2	15	77.5	3.9	16
9	63.0	0.0	1	51.0	15.0	3
10	40.0	0.0	1	40.0	0.0	2

Note that success corresponds to the number of cases the method return a value.

Supplementary Table S5

Leadership over the entire length of the observation (column 2 and 3) and using the intermittent causality detection method (column 4 and 5) along with kinematics of the birds involved in individual dataset of Cliff Swallow.

Data	$netTE^{1\to 2}$	Leader	time(s)	Switching (bird $i \to j$)	Speed	(m s^{-1})	Acceleration	(m s^{-2})	Rate of turns	(rad s^{-1})
					bird1	bird2	bird1	bird2	bird1	bird2
1	0.003	–	–	–	6.9	7.4	9.3	11.6	1.3	1.5
2	0.041	1	–	–	2.9	3.6	8.9	15.5	2.6	3.6
3	0.01	1	–	–	5.4	6.0	7.6	7.0	1.3	1.1
4	0.011	1	3	$2\to 1$	6.0	6.4	11.1	13.2	1.7	1.9
5	0.004	–	–	–	6.6	7.0	18.8	17.2	2.7	2.3
6	0.004	–	–	–	6.3	7.0	12.7	13.3	2.0	1.8
7	0.039	1	2.25	$2\to 1$	5.4	5.8	13.1	16.3	2.3	2.5
8	-0.007	–	0.75	$2\to 1$	3.4	3.6	10.6	9.5	2.9	2.5
9	0.014	1	–	–	9.5	11.8	18.1	20.2	1.8	1.7
10	0.011	1	0.75	$2\to 1$	6.1	6.2	7.4	7.6	1.1	1.2
11	0.016	1	3.5	$1\to 2$	5.5	6.3	13.4	15.1	2.5	2.3
12	-0.057	2	–	–	10.4	10.4	22.4	20.9	2.1	1.7
13	0.001	–	–	–	5.7	5.9	9.4	10.3	1.8	1.9
14	0.032	1	–	–	8.1	8.9	14.3	16.4	1.7	1.8
15	0.017	1	–	–	5.8	5.8	8.5	10.4	1.4	1.6
16	0.014	1	–	–	8.0	7.4	9.5	7.7	1.1	1.0
17	0	–	3	$1\to 2$	5.1	5.3	5.8	7.1	1.1	1.3
18	-0.005	–	0.75	$2\to 1$	6.2	5.8	7.7	8.8	1.2	1.4
19	-0.016	2	–	–	12.3	13.6	40.7	42.4	3.2	3.2
20	0	–	–	–	8.7	8.7	4.7	6.9	0.5	0.8
21	0.029	1	3.75	$2\to 1$	8.4	8.8	13.0	13.1	1.5	1.5
22	0.003	–	–	–	6.5	7.7	12.5	15.0	1.8	2.0
23	0	–	–	–	4.9	5.4	7.9	11.8	1.5	2.3
24	-0.002	–	0.75	$2\to 1$	7.9	8.2	5.9	7.6	0.8	1.0
25	0.022	1	–	–	4.8	5.4	12.9	11.9	2.8	2.3
26	-0.003	–	–	–	6.5	7.7	6.5	8.2	0.8	0.9
27	0.018	1	0.75	$1\to 2$	6.0	5.7	19.7	14.9	3.5	2.6
28	0.023	1	0.75	$2\to 1$	7.1	7.0	15.3	18.6	2.0	2.5
29	0.006	–	–	–	7.9	7.8	12.1	11.3	1.4	1.2
30	0.047	1	0.75	$2\to 1$	12.0	12.6	12.9	12.6	1.0	0.9
31	0.074	1	–	–	5.4	5.2	11.2	13.2	1.8	2.1
32	0.03	1	–	–	6.1	6.4	13.5	14.4	2.1	2.1
33	0.017	1	–	–	5.5	5.7	13.9	12.1	2.3	2.0
34	0	–	3	$1\to 2$	5.2	5.4	4.4	4.5	0.8	0.7
35	0.034	1	0.75	$1\to 2$	5.6	5.6	10.4	12.3	1.6	1.8
36	0.017	1	–	–	3.7	4.2	9.8	11.7	2.3	2.4
37	0.036	1	0.75	$1\to 2$	5.3	5.5	11.5	11.6	1.9	1.9
38	-0.002	–	–	–	4.9	4.9	9.3	10.2	1.6	1.8
39	0.042	1	0.75	$2\to 1$	7.9	8.1	11.7	16.0	1.4	1.9
mean	$0.013^{\star}$	–	–	–	6.5	6.8	11.7	12.8	1.8	1.8
se	0.003	–	–	–	0.3	0.4	1.0	1.0	0.1	0.1

Supplementary Video 1 Tandem birds flight with consistent leadership.

Supplementary Video 2 Tandem flight with switching leadership.
